# Modulation of Corticospinal Excitability during Acquisition of Action Sequences by Observation

**DOI:** 10.1371/journal.pone.0037061

**Published:** 2012-05-16

**Authors:** Masanori Sakamoto, Noriyoshi Moriyama, Nobuaki Mizuguchi, Tetsuro Muraoka, Kazuyuki Kanosue

**Affiliations:** 1 Faculty of Sport Sciences, Waseda University, Tokorozawa, Saitama, Japan; 2 Department of Physical Education, Faculty of Education, Kumamoto University, Kumamoto, Japan; 3 Graduate School of Sport Sciences, Waseda University, Tokorozawa, Japan; 4 College of Economics, Nihon University, Tokyo, Japan; Harvard Medical School, United States of America

## Abstract

Excitability of the corticospinal pathway increases during observation of an action. However, how corticospinal excitability changes during observation of sequential actions in the course of acquiring novel skills (observational learning) remains unexplored. To investigate this, we used a previously unpracticed sequence of ten hand postures. Participants were asked to repeat observation and replication of the sequence. This block of observation and replication was repeated 5 times. During observation of a given hand posture (OK sign), motor-evoked potentials (MEPs) elicited by transcranial magnetic stimulation were recorded from hand muscles. In experiment 1, the OK sign appeared in the 9th position of the sequence. Almost all participants could replicate the OK sign only at the 5th block of the experiment. MEP amplitude was greater than that in the control, and decreased with the stages. This suggested that during observational learning of sequential hand postures MEP changed with the progress of the learning. To evaluate this idea, we performed two additional experiments. In experiment 2, the OK sign appeared in the 2nd position. Almost all participants replicated the OK sign even in the 1st block. The MEP amplitude did not change across stages. In experiment 3, the OK sign appeared in the 9th position, but the order of other signs was randomized in every stage. Many participants were not able to replicate the OK sign even during the 5th block of the experiment. The MEP amplitude did not change across stages. These results suggest that: (1) During observational learning modulation of corticospinal excitability is associated with the learning process. (2) Corticospinal excitability decreases as learning progresses.

## Introduction

Although most motor skills are acquired through physical practice, it is generally believed that observing an action performed by others is important in the improvement of the observer's motor skills [1]. When humans try to perform untrained and novel actions, typically they first observe the actions by experts, so as to aid in the incorporation of critical elements of the actions into their motor repertoire [2]. Indeed, there is behavioral evidence that observation of movements alone improves specific motor performance indices, such as reaction time, movement direction and movement trajectory [3]–[6].

The behavioral improvements follow excitability changes in the motor systems of the brain. Observation of an action modulates excitability of the corticospinal pathway, as was investigated by measuring the motor-evoked potential (MEP) to stimulation of the primary motor cortex using transcranial magnetic stimulation (TMS). For example, Fadiga et al. [7] showed that when humans observed a grasping action performed by others, the MEP of the hand muscles increased. The increases in MEP were largely specific to the muscles involved in the observed action [7]–[10]. Furthermore, a strict phase coupling between changes in MEP and the dynamics of the observed action was noted [11], [12]; during observation of a grasping action, MEP in finger muscles increased during the finger aperture phase and decreased during the finger closure phase. Clearly, visual information is processed and forwarded in such a way that it can alter signals in the motor pathways that control movement.

Modulation of MEP during action observation depends on the observers' long-term experience. Aglioti et al. [13] reported an increase in MEP in elite basketball players when they observed basketball shots, while no increase in MEP was shown when they observed soccer kicks. In contrast, MEP of non-athletes was modulated during observation of the actions of both sports, which was postulated to indicate nonspecific activation of the motor system. Molnar-Szakacs et al. [14] demonstrated a culture specific modulation of MEP. Euro-American participants showed a large MEP during the observation of classic American gestures performed by an American actor as compared with those performed by a Nicaraguan actor.

However, how the MEP changes during observation of unpracticed action in the course of acquiring novel skills (observational learning) remains unexplored. In the present study, to examine this question, we used unpracticed sequences of 10 different hand postures. We focused on the learning process of the sequences rather than individual hand postures. Without practice, the participants were capable of making each individual posture without difficulty. After only using observation to learn the sequence of hand postures, they were required to actually perform the newly learned sequence. In the experiment 1, we investigated the modulation of MEP amplitude during observation of the OK sign. To confirm and extend the findings obtained from experiment 1, we performed experiments 2 and 3. In these experiments, the presentation manner of the OK sign was altered. This allowed us to investigate how acquisition of the OK sign influenced changes in MEP during the course of observational learning.

## Materials and Methods

### Participants

Twenty-nine right-handed volunteers aged 22 to 34 years (18 males, 11 females), naive to the purpose of the experiments, participated in the study. All participants had normal results on physical and neurological examinations and gave written, informed consent. This study was approved by the Human Research Ethics Committee of the Faculty of Sport Sciences, Waseda University. The experiments were conducted in accordance with the Declaration of Helsinki.

### Recordings

The electromyographic responses (EMG) were recorded from the left first dorsal interosseous (FDI), opponens pollicis (OP) and the abductor digiti minimi (ADM) muscles with disposable Ag-AgCl electrodes placed over the belly of muscles. The EMG signal was amplified (MEB-2216, Nihonkoden, Japan) and bandpass filtered between 5 and 1500 Hz. All signals were converted into digital data via an A/D converter system at a sampling rate of 3 kHz and recorded for later analysis.

### Transcranial magnetic stimulation

TMS was delivered by the magnetic stimulator (Magstim 200, Magstim Co., UK) with a figure 8-shaped coil (each diameter 70 mm). Single-pulse TMS was delivered to the right hemisphere and the MEPs evoked in the left FDI, OP and ADM were recorded. Optimal scalp position was determined utilizing a slightly suprathreshold stimulus intensity. The coil was moved over the right hemisphere so that the position over the scalp at which a maximal MEP amplitude was elicited in the FDI could be determined. With this coil position, it was possible to record a stable signal from OP and ADM in all participants. The optimal position of the coil was marked on the scalp with a pen so that correct coil placement could be ensured throughout the experiment. The coil was placed tangentially to the scalp with the junction region pointing backwards and laterally at a 45 deg angle away from the mid-line, approximately perpendicular to the line of the central sulcus, inducing a posterior-anterior current in the brain. We chose this orientation because motor threshold is minimum when the induced electrical current in the brain flows approximately perpendicular to the line of the central sulcus [15], [16]. Stimulus intensity was set at 120% of the resting motor threshold (RMT), defined as the minimum stimulus intensity that produced EMG responses greater than 50 µV in FDI in at least five out of ten trials.

### Tasks

Participants sat comfortably in an arm chair with the left forearm in a prone position. The participants' left elbow angle was flexed (at about 120°). A screen was set 100 cm in front of the participants who performed 3 different tasks. The details were as follows:

#### Control task

**Figure 1:**
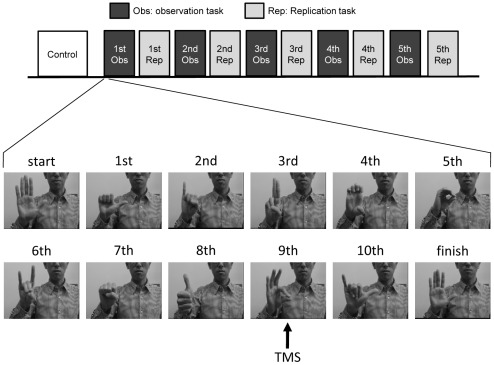
The design of experiment 1. The video clip showed a sequence of 10 different hand postures of the right hand during an observation task. This was repeated 12 times in each block of the observation task. Transcranial magnetic stimulation was applied when participants observed the OK sign (in the 9th position) with the thumb and index finger. The observation task and the replication task were alternately repeated 5 times.

Participants were asked to observe a static and upright right hand picture (“start” sign in Fig. 1) presented on the screen. During observation of the hand picture, ten TMS were applied with an interpulse interval of about 15 s.

#### Observation task

Participants were asked to observe a video clip, which included the right hand of a person consecutively performing 10 different hand postures. The video clip took 14 sec, and depicted the postures in a fixed order (Fig. 1). Participants were instructed to learn the sequence, starting with the first posture, without any movement of the hand. All hand postures were meaningless to the participants. A video of the sequence of hand postures was continuously repeated 12 times in one block of the observation task. TMS was applied when participants observed the posture with a closed thumb and index finger (“OK sign”, the 9th posture in Fig. 1). TMS was triggered by a negative logic pulse generated by a photodiode sensor (PLDM-10, Sankei, Co., LTD., Tokyo) that detected the time of the contact between the thumb and index finger. The sensor was positioned on the screen in front of the participants.

#### Replication task

After each block of the observation task, participants were asked to perform one replication of the sequence of 10 hand postures that were observed during the observation task (Fig. 1). Participants used their left hand in a mirror-image fashion. In the present study, the mirror configuration was selected because there is a natural tendency to imitate in the mirror configuration [17], [18]. Participants were instructed to try to replicate the hand postures utilizing the same pace as was presented in the video-clip. Participants' actions were recorded with a video camera.

### Experimental procedures

#### Experiment 1

Ten participants performed this experiment. Initially, a block of control tasks was made: MEPs were recorded when participants viewed a static hand picture presented on the screen center with no muscle activity. Then the participants alternately repeated observation and replication tasks 5 times (Fig. 1). Of the 12 video-clip presentations made in each block, ten involved TMS pulses. To suppress the participants' anticipation for TMS, 2 catch trials were randomly made. In these, TMS was applied at the 9th posture (OK sign). A total of 50 TMS pulses were applied.

#### Experiment 2

Nine participants were involved in this experiment. The experimental procedure was the same as that in experiment 1 except the presentation order of hand postures was reversed. TMS was applied when the 2nd posture (OK sign) was presented. This 2nd posture was identical to the 9th posture of experiment 1.

#### Experiment 3

Ten participants took part in this experiment. The experimental procedure was the same as in experiments 1 and 2 except for the method of presentation of hand postures during the observation task. Ten hand postures were presented that were identical to those used in experiments 1 and 2, but with the order of postures being changed in every block. However, the 9th posture in all blocks was the same as the 9th posture presented in experiment 1 (OK sign). TMS was applied when the 9th posture was shown in every block.

### Data analysis

EMG signals were recorded from 100 ms before to 100 ms after the TMS. An average prestimulus EMG activity was obtained by calculating the root mean square for 100 ms before the TMS for each block. To estimate corticospinal excitability, the peak to peak amplitude of the MEP was measured and normalized with respect to the MEP obtained during the control task. MEPs were averaged across the 10 TMS trials in each block.

Because we executed the present study in successive steps, experiments 2 and 3 were performed after the results were obtained for the preceding experiment. Therefore, direct comparisons among the three experiments were not performed. Instead, a separate statistical analysis was made for each of the three experiments. During the replication task we recorded participants' actions with a video-camera. The number of correct responses in each replication task was obtained from a video recording of the participants' actions while performing the task. If the participants mistook the hand action sequences or if they were not able to replicate the correct postures within 2 sec after the previous hand posture, these responses were counted as incorrect. We defined the number of correct responses as the number of consecutive times the participant replicated hand postures from the first one.

For changes in MEP amplitude and prestimulus EMG across the block number, one-way repeated measures analysis of variance (ANOVA) was performed. For post-hoc comparisons, multiple pair-wise tests with Bonferoni's correction were performed. To investigate whether the MEP sizes were significantly increased relative to the control runs, multiple comparisons were conducted using Dunnett's test. For evaluation of the number of replicated hand positions during the replication task, the Friedman test was performed. For the number of participants who correctly replicated the OK sign during the replication task, Cochran's test was performed. Data were expressed as the mean ± one standard error. Significance was set at p<0.05 except for the post-hoc comparisons. For the post-hoc comparisons, significance was set at p<0.005.

## Results

### Experiment 1

The test TMS intensity was 64.9±9.0% of the maximal output of the magnetic stimulator. Prestimulus EMG activities in the FDI, OP and ADM were not different across all blocks of the observation task (FDI: F(4, 36) = 0.76, p>0.05, OP: F(4, 36) = 2.34, p>0.05, ADM: F(4, 36) = 1.78, p>0.05).

**Figure 2:**
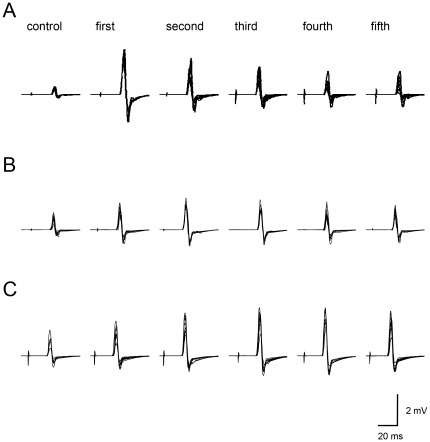
Typical recordings of MEPs in FDI during experiment 1 (A), 2 (B) and 3 (C). These waveforms were obtained from three different participants. Ten traces were superimposed for each waveform.

**Figure 3:**
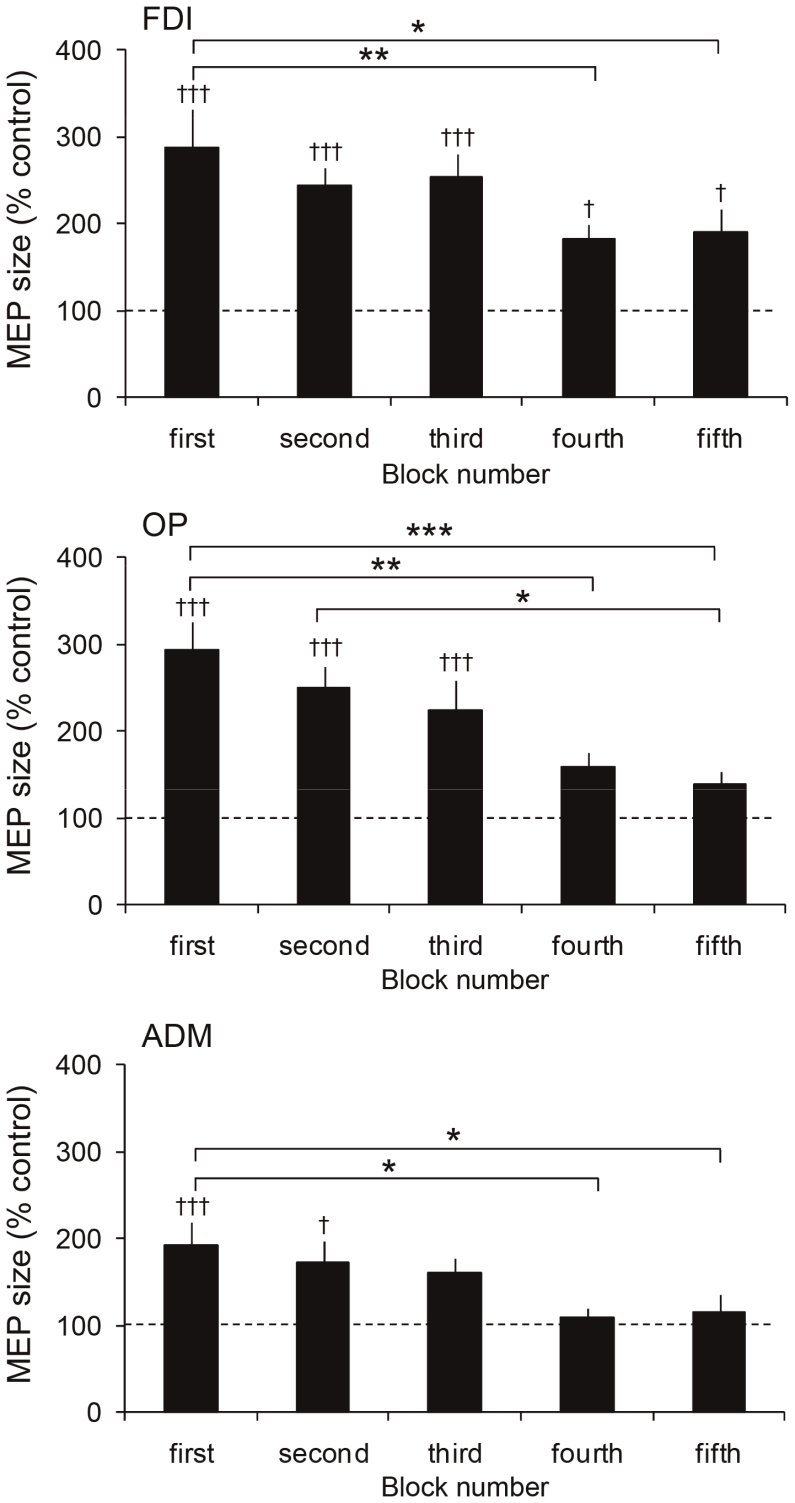
Mean MEP size in three muscles during the observation task in experiment 1. Values on the ordinate indicate MEP size as a percentage of those obtained in the control task. Data are represented as the mean ± one SE. Asterisks indicate significant differences between blocks. Daggers indicate significant differences from the control value. * p<0.05, ** p<0.01, † p<0.05, ††† p<0.001.

Figure 2A shows typical recordings of MEPs in the FDI obtained from a single participant. Figure 3 illustrates group means of MEPs during the observation task. For the FDI, one-way repeated measures ANOVA demonstrated a significant main effect for the block number (F(4, 36) = 5.11, p<0.01). The MEPs in the fourth and fifth blocks were significantly smaller than those in the first block (fourth: p<0.001, fifth: p<0.005). In addition, MEPs across all blocks of the observation task were significantly greater than those observed during the control task (first, second and third: p<0.001, fourth and fifth: p<0.05). In the first block, the MEP was about three times as large as that during the control task. The enhancement of MEP value decreased across the block of the observation task. In the final block, MEP reached levels that were about the twice that of the control.

Similar tendencies were obtained from the OP and ADM. For the OP, one-way repeated measures ANOVA demonstrated a significant main effect for the block number (F(4, 36) = 8.67, p<0.001). MEPs in the fourth and fifth blocks of the observation task significantly decreased as compared to those of the first block (fourth: p<0.001, fifth: p<0.0001). MEP in the fifth block was also smaller than those of the second block (p<0.005). Furthermore, MEPs in the first, second and third blocks were significantly greater than those observed in the control task (first and second: p<0.001, third: p<0.01). For the ADM, one-way repeated measures ANOVA demonstrated a significant main effect for block number (F(4, 36) = 4.23, p<0.01). MEPs in the fourth and fifth blocks significantly decreased as compared to those in the first block (both p<0.005). MEPs in the first and second blocks were significantly greater than those in the control task (first: p<0.01, second: p<0.05).

For the replication task, the participants exhibited pronounced learning effects across the five blocks (p<0.001, Table 1). The number of replicated postures in the correct order in the first block was 2.2±0.5. In the fifth block it increased to 8.7±0.7. The number of participants who replicated the OK sign significantly increased across the trials (p<0.05, Table 2). Although only 2 out of the 10 participants were able to replicate the OK sign by the fourth block of the test, 7 participants correctly replicated it in the fifth block.

**Table 1:** The number of correct responses during the replication task.

	first	second	third	fourth	fifth
Exp. 1	2.2±0.5	5.3±0.7	6.9±0.8	6.2±0.9	8.7±0.7
Exp. 2	2.8±0.6	4.7±0.8	8.1±0.8	7.8±1.0	9.7±0.3
Exp. 3	3.1±0.9	5.8±1.0	5.8±0.9	5.6±1.1	6.8±1.1

**Table 2:** The number of participants who correctly replicated the OK sign during the replication task.

	first	second	third	fourth	fifth
Exp. 1 (9th)	0	1	3	2	7
Exp. 2 (2nd)	6	10	10	10	10
Exp. 3 (9th)	0	2	2	2	4

There was no significant correlation between MEP amplitudes and the number of replicated postures (p>0.05).

### Experiment 2

The test TMS intensity was 60.2±7.3% of the maximal output of the magnetic stimulator. Prestimulus EMG activities in the FDI, OP and ADM were not different across all blocks of the observation task (FDI: F(4, 32) = 2.34, p>0.05, OP F(4, 32) = 1.96, p>0.05, ADM: F(4, 32) = 1.65, p>0.05).

**Figure 4:**
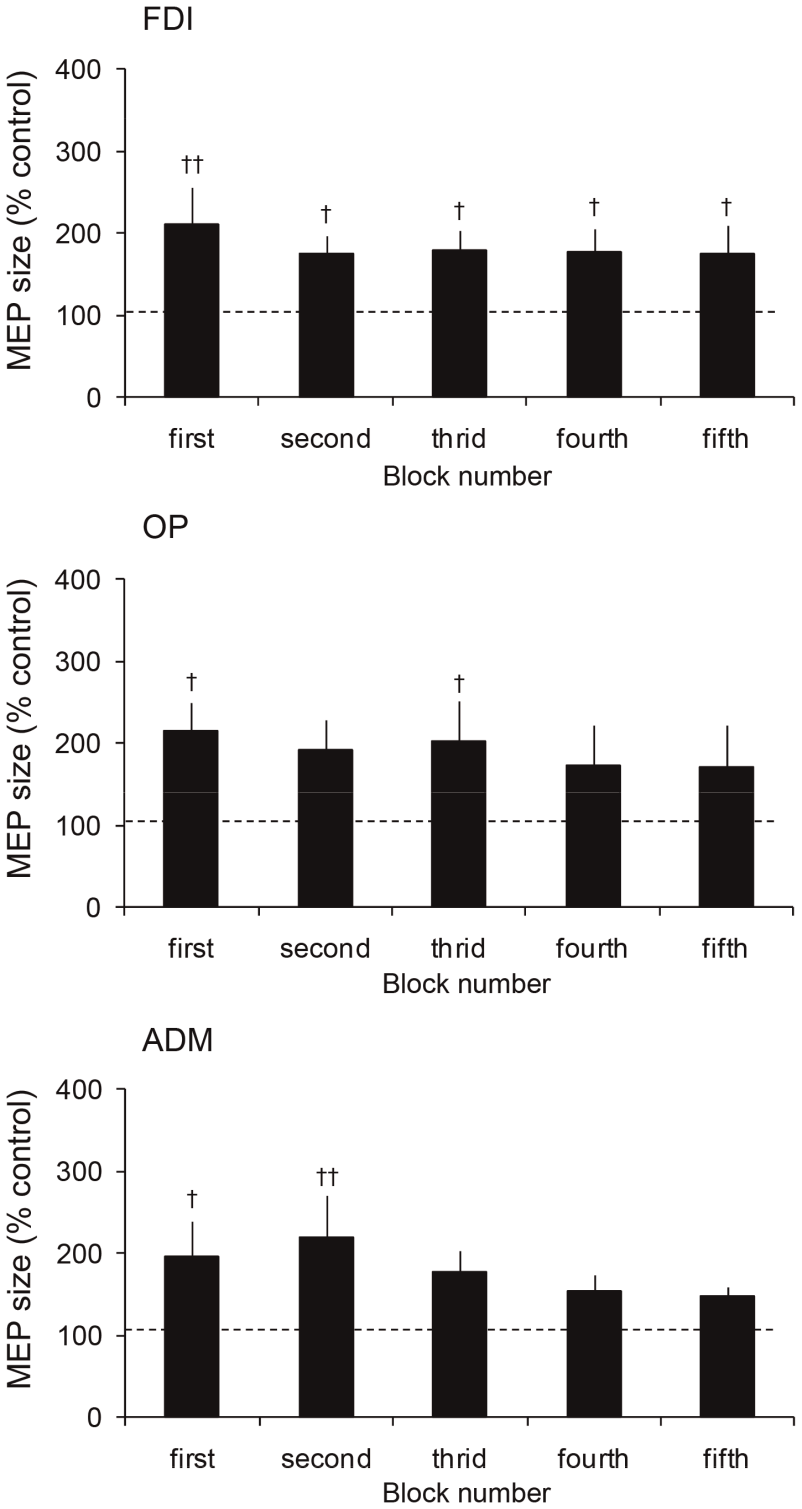
Mean MEP size during the observation task in experiment 2. Values on the ordinate indicate MEP size as a percentage of those obtained in the control task. Data are represented as the mean ± one SE. Daggers indicate significant differences from the control value. † p<0.05, †† p<0.01.

Figure 2B shows typical recordings of MEPs in the FDI obtained from a single participant. Figure 4 illustrates group means of MEPs during the observation task. For the FDI, one-way repeated measures ANOVA demonstrated no significant main effect for block number (F(4, 32) = 0.79, p>0.05). MEP in all blocks were significantly larger than those of the control task (first: p<0.01, second, third, fourth and fifth: p<0.05). In the first block, the MEP was about twice as large as those of the control task. The magnitude of the MEP was not altered along the blocks. For the OP, one-way repeated measures ANOVA demonstrated no significant main effect for block number (F(4, 32) = 0.72, p>0.05). MEPs in the first and third blocks were significantly larger than those of the control task (both p<0.05). For the ADM, one-way repeated measures ANOVA demonstrated no significant main effect for block number (F(4, 32) = 1.48, p>0.05). MEPs in the first and second blocks were significantly larger than those of the control task (first: p>0.05, second: p>0.01).

For the replication task, a significant main effect for block number was observed (p<0.001, Table 1). In the first block of the replication task, the number of replicated postures in the correct order, from the first one, was 2.8±0.6. Most of the participants were able to perfectly replicate the 10 postures in the fifth block. The number of participants who replicated the OK sign was not significantly increased across trials (p>0.05, Table 2). Six out of the 10 participants replicated the OK sign in the first block. All the participants were already able to replicate the OK sign by the second block.

There was no significant correlation between MEP amplitudes and the number of replicated postures (p>0.05).

### Experiment 3

The test TMS intensity was 60.4±8.1% of the maximal output of the magnetic stimulator. Prestimulus EMG activities in the FDI, OP and ADM were not different across all blocks of the observation task (FDI: F(4, 36) = 1.42, p>0.05, OP: F(4, 36) = 0.49, p>0.05, ADM: F(4, 36) = 0.63, p>0.05).

**Figure 5:**
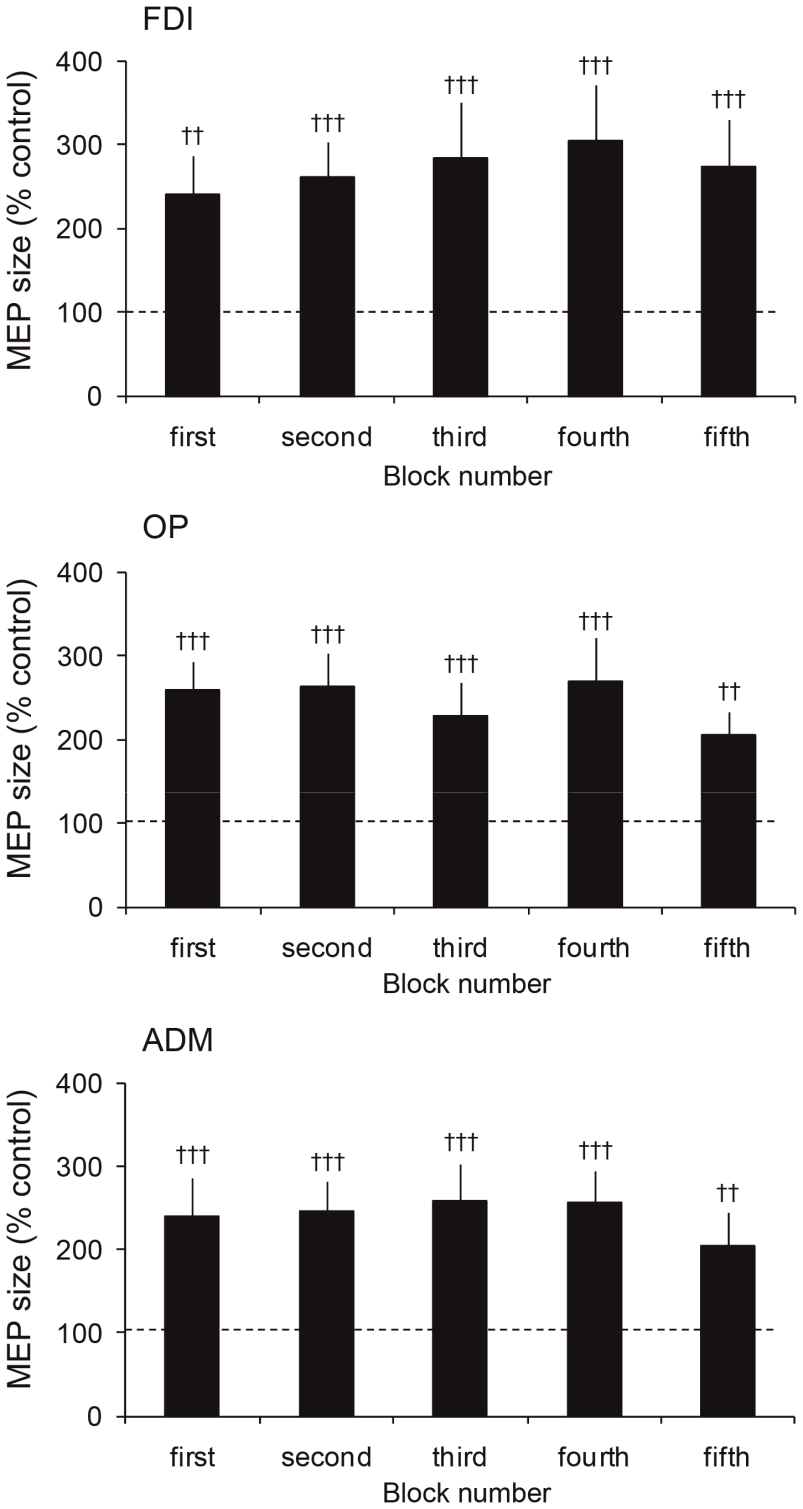
Mean MEP size during the observation task in experiment 3. Values on the ordinate indicate MEP size as a percentage of those obtained in the control task. Data are represented as the mean ± one SE. Daggers indicate significant differences from the control value. †† p<0.01, ††† p<0.001.

Figure 2C shows typical recordings of MEPs in the FDI obtained from a single participant. Figure 5 illustrates group means of MEPs during the observation task. For the FDI, MEPs were not changed across all blocks of the observation task (F(4, 36) = 2.05, p>0.05), and were maintained at about 3 times that of the control task until the final block (first: p<0.001, second, third, fourth and fifth: p<0.001). Likewise, for the OP and ADM, a one-way repeated measures ANOVA demonstrated no significant main effect for block number (OP, F(4, 36) = 3.59, p>0.05; ADM, F(4, 36) = 1.43, p>0.05). MEPs in all blocks of the observation task were significantly larger than those of the control task (first, second, third and fourth: p<0.001, fifth: p<0.01).

For the replication task, the participants exhibited learning effects across the five blocks (p<0.05, Table 1). In the first block the number of replicated signs, in order, from the first attempt was 3.1±0.9. Although the number of replicated postures had increased to 6.8±1.1 in the fifth block, most of the participants were still not able to perfectly replicate the whole set of 10 consecutive postures. The number of participants who replicated the OK sign was not significantly changed across the trials (p>0.05, Table 2). Only 4 out of the 10 participants were able to correctly replicate the OK sign in the fifth block.

There was no significant correlation between MEP amplitudes and the number of replicated postures (p>0.05).

## Discussion

In this study we investigated the MEP modulation during the course of acquiring a previously unpracticed sequence of hand postures by observation. To obtain the requisite information we utilized three different experiments. Participants were asked to observe 10 different hand postures (observation task), and then replicate the observed 10 postures, in the same order, starting with the first one (replication task). When participants observed the OK sign, the MEP of the hand muscles was measured. The presentation order of the OK sign was different across the three experiments. In the experiment 1, the OK sign was presented in the 9th position. In the first block of the observation task the MEP magnitude was about three times as large as that of the control task, and its magnitude decreased to about the twice that of the control in the final (fifth) block. In the replication task, all the participants were not able to replicate the OK sign in the first block. However, seven of the 10 participants could replicate the OK sign by the final block. These results suggested that the MEP was large when observational learning was incomplete, and decreased as learning progressed.

To confirm and extend this observation we performed experiments 2 and 3. In experiment 2, we used a video-clip composed of the same 10 hand postures as in experiment 1, but the presentation order was reversed. The earlier presentation of the OK sign allowed participants to memorize the sign easily. Thus, all the participants were able to replicate the OK sign by the second block of the replication task. Under this condition, the MEP did not change across blocks. Although the MEP was elevated, it was only about the twice that of the control.

For experiment 3, we used a video-clip composed of the same 10 hand postures as those used in experiments 1 and 2, but with the presentation order changed in every block except for the OK sign. The OK sign was always presented in the 9th position in all blocks, just as was done in experiment 1. Therefore, a difficulty in acquisition of the OK sign was maintained at a high level across all blocks. Indeed, although the total number of replicated postures slightly increased across blocks, the number of participants who correctly replicated the OK sign was not significantly increased. This indicated that the observation task in experiment 3 made it extremely difficult to replicate the OK sign. In this condition, the MEP amplitude in all blocks was larger than those of the controls, and did not change across blocks. Thus, the results of the experiment 2 and 3 lend credence to the earlier supposition that the MEP amplitude does not change once the learning has been completed or it is still incomplete.

Is the MEP amplitude related to the outcome of replication task? Apparently not, since there was not a significant correlation between the MEP size and the number of postures correctly replicated across all the experiments. Rather, MEP amplitudes are likely to reflect the degree of the observer's effort at the time when the TMS is administered.

### Implications for neural mechanisms

In experiment 1, the MEP in the last block decreased as compared to that of the first block. This finding is inconsistent with the MEPs that occurred during observation in our previous study [19]. In that study the MEP amplitude did not change when participants repeated observation of a pinching action with the thumb and index finger. The MEP amplitude rather gradually increased when participants alternately repeated observation of the pinching action and execution of the same action. The discrepancy between the two experiments might be caused by a difference in the purpose of the action observation. In the present study participants were asked to observe the hand postures to learn the sequence and to replicate it after the period of observation. In contrast, in our previous study participants were instructed just to observe a pinching action. They did not have specific purpose for learning the particular posture. In this vein, Clark et al. [20] demonstrated that having a different purpose for a particular action observation led to a different modulation of the MEP. The MEP recorded during the observation of a hand action that was to be later imitated was enhanced to a greater degree than the MEP that occurred during mere observation of the same action. The MEP modulation that occurred in the present study was likely caused by neural mechanisms that work specifically for replicating action by utilizing observation.

What neural elements contributed to the MEP modulation in the present study? One candidate might be the mirror neuron system (MNS). The MNS is thought to be located in the premotor and parietal areas, and is activated not only by an action but also when the same action is observed while being performed by others [2], [21], [22]. The MNS might transform sensory information into motor representation. When humans observed a hand action performed by others, MEP amplitude for their hand muscles increased [7], [9]. In addition, modulation of MEP amplitude during observation of an action was dependent upon the dynamics of the observed action [11], [12]. These modulations of MEP would likely be produced by the MNS. Activation of the premotor MNS during observation of an action could produce the enhancement of the MEP via cortico-cortical projections from the premotor cortex to the primary motor cortex [23], [24]. Indeed, when the ventral premotor cortex was inactivated by low-frequency, repetitive TMS, the increase in the MEP that occurred during observation of hand action disappeared [25].

Buccino et al. [26] and Vogt et al. [27] investigated brain activities in guitar experts during observation of guitar chords they would have to imitate. Activity of the MNS was enhanced to a greater degree during observation of novel chords than for those that they had had previous experience with. These modulations of MNS activities during observational learning are similar to those of the MEP obtained in the present study. Thus, the MEP modulations when acquiring novel action sequences by observation without overt actions could be caused by activity changes in the MNS.

Modulation of the MEP showed almost the same pattern among FDI, OP and ADM muscles. For the MEP of FDI and OP, the modulations appear logical, since these two muscles are involved in making the OK sign. MEP modulations during action observation have been shown to be restricted to the muscles which would be activated during actual execution of the observed movement [8], [9], [19]. However, the ADM is not activated in the production of the OK sign. Although we cannot provide a clear explanation for the discrepancy, we speculate that it might be caused by a less obvious aspect of the task. In previous studies, participants were asked to observe simple and repeated actions [8], [9], [19]. This lead to selective activation of the hand motor area involved in the observed action. In the present study, participants were asked to observe the sequence of various hand postures for replication. In this case, when learning was incomplete, individual cortical motor areas innervating different hand muscles could not be activated separately so as to be related to a specific action, because incomplete learning means that the specific, involved muscles are not yet fixed in the brain. Thus, hand motor areas might be widely activated when participants were still learning action sequences by observation. Further studies are necessary to test this possibility.

Action observation and imitation are expected to be a new tool for neurorehabilitation [28], [29]. Recovery of motor function after stroke includes several stages: action observation, motor imagery and motor execution. Action observation might be most efficient at an early stage of motor recovery. Thus, understanding the neural mechanisms of observational learning could contribute to the development of new methods for the recovery of motor function in such patients. In addition, our findings help form an understanding of the basis for the motor learning that is involved in sports and complex actions [30]. When children acquire novel actions, their learning is facilitated by observing or imagining the actions performed by others. Elucidation of the neural mechanisms involved in observational learning is expected to increase the effectiveness of training methods utilized for the acquisition of complex motor sequences. Further studies are needed to fully achieve these goals.

In conclusion, the present study demonstrated that during observational learning of sequential hand postures the MEP was enhanced at earlier stages when the learning was incomplete. While the neural mechanism underlying this modulation remains to be determined, the MNS could contribute to the modulation of the MEP in the course of acquiring action sequences by observation.
